# Factors influencing long-term outcomes in fibrotic interstitial lung disease (F-ILD) diagnosed through multidisciplinary discussion (MDD): a prospective cohort study

**DOI:** 10.1186/s40001-024-01673-2

**Published:** 2024-01-30

**Authors:** Yu-Wan Liao, Ming-Cheng Liu, Yu-Cheng Wu, Chiann-Yi Hsu, Wen-Nan Huang, Yi-Hsing Chen, Pin-Kuei Fu

**Affiliations:** 1 Integrated Care Center of Interstitial Lung Disease, Taichung Veterans General Hospital, Taichung, 40705 Taiwan; 2 Division of Allergy, Immunology and Rheumatology, Department of Internal Medicine, Division of Allergy, Taichung Veterans General Hospital, Taichung, 40705 Taiwan; 3 Department of Radiology, Taichung Veterans General Hospital, Taichung, 40705 Taiwan; 4 Department of Critical Care Medicine, Taichung Veterans General Hospital, Taichung, 40705 Taiwan; 5 Biostatistics Task Force, Department of Medical Research, Taichung Veterans General Hospital, Taichung, 40705 Taiwan; 6 Department of Post-Baccalaureate Medicine, College of Medicine, National Chung Hsing University, Taichung, 40200 Taiwan; 7 Division of Clinical Research, Department of Medical Research, Taichung Veterans General Hospital, 1650 Taiwan Boulevard Sect. 4, Taichung, 407219 Taiwan

**Keywords:** Antifibrotic agents, GAP score, Idiopathic pulmonary fibrosis, Interstitial lung disease, UIP

## Abstract

**Background:**

The diagnostic process for fibrotic interstitial lung disease (F-ILD) is notably intricate, necessitating a multidisciplinary discussion to achieve consensus based on both clinical and radiological features. This study investigated the shared and distinctive long-term mortality predictors among the two primary phenotypes of F-ILD, namely idiopathic pulmonary fibrosis (IPF) and connective tissue disease-associated interstitial lung disease (CTD-ILD).

**Methods:**

We included patients with F-ILD diagnosed from December 2018 to December 2019 and conducted follow-up assessments until February 2023. Age, gender, usual interstitial pneumonia (UIP) pattern, gender–age–physiology (GAP) score, modified Medical Research Council (mMRC) dyspnea score, antifibrotic agent use, pulmonary function test parameters, and six-minute walking test (6MWT) parameters were recorded at baseline and used as mortality predictors in a multivariate Cox regression model.

**Results:**

We enrolled 104 ILD patients. The survival rate of non-IPF patients was more than twice that of IPF patients (78.9% vs. 34%, *p* < 0.001), and the survival rate of patients with a GAP score of 0–2 was more than twice that of patients with a score of > 2 (93.2% vs. 36.6%, *p* < 0.001). Older age, male gender, definite UIP pattern, higher GAP score, higher mMRC dyspnea score, lower forced expiratory volume in one second/forced vital capacity (FEV1/FVC), shorter 6MWT distance, and lower initial and final SpO2 were also associated with higher long-term mortality (*p* < 0.05). In multivariable analysis, only a GAP score of > 2 (hazard ratio [HR]:16.7; 95% confidence interval [CI] 3.28–85.14; *p* = 0.001) and definite UIP pattern (HR: 4.08; 95% CI 1.07–15.5; *p* = 0.039) were significantly associated with overall mortality.

**Conclusion:**

The long-term mortality rate of IPF patients was higher than that of CTD-ILD patients**.** The GAP score and UIP patterns were significant mortality predictors for both IPF and CTD-ILD patients.

**Supplementary Information:**

The online version contains supplementary material available at 10.1186/s40001-024-01673-2.

## Background

Fibrotic interstitial lung diseases (F-ILDs) are a miscellaneous group of disorders with the hallmark of lung scaring or fibrosis [1], which compromises the respiratory system’s oxygenation and diffusing capacity for carbon monoxide (DLCO) [2]. There are over 200 F-ILDs, many of which share clinical, radiological, and pathological characteristics. Major etiologies of F-ILDs include idiopathic conditions like idiopathic interstitial pneumonia and idiopathic pulmonary fibrosis (IPF) [3], as well as connective tissue disease-associated ILDs (CTD-ILDs) like those linked to rheumatoid arthritis [4].

Idiopathic pulmonary fibrosis is one of the most aggressive ILDs and currently lacks a definitive cure. In IPF, excessive fibroblast proliferation leads to increased deposition of extracellular matrix proteins, especially collagen. This exacerbates recurrent scarring and fibrosis of the lung parenchyma, increasing pulmonary rigidity. Consequently, the ability to take up oxygen and exchange gases is impaired. Prognosis after an IPF diagnosis is poor, with a median survival of 2–3 years. The etiology of IPF remains uncertain, and there are no effective treatments that significantly improve patient outcomes [5]. Furthermore, the nosological validity of IPF itself is debated. Some advocate splitting it into different subtypes with unique responses to tailored therapies, while others promote lumping IPF with other advancing fibrotic lung diseases that share common pathogenetic mechanisms and disease progression [6]. These advancements suggest a trend towards precision-based methodologies in comprehending and treating ILD, emphasizing individualized therapeutic strategies and nuanced classifications. [7].

Patients with IPF often face significant comorbidities that impact their overall health and quality of life. Depression is a common comorbidity, as highlighted by Tzouvelekis et al. [8], which emphasizes its detrimental impact on the wellbeing of individuals with IPF. Additionally, there is a notable association between IPF and an increased risk of lung cancer, as detailed by Karampitsakos et al. [9] and further corroborated by the DIAMORFOSIS survey [10]. These studies underscore the need for comprehensive care strategies that address not only the pulmonary condition but also the accompanying comorbidities to improve the overall prognosis and quality of life for patients with IPF.

Given the extensive pathological and prognostic diversity of ILDs combined with clinical and radiological mimicry, multidisciplinary discussion (MDD) between experts, including pulmonologists, radiologists, pathologists, and rheumatologists, is critical for accurate ILD diagnosis as highlighted by the American Thoracic Society and European Respiratory Society since 2001 [11]. Recently published guidelines and epidemiological studies further emphasize the necessity and value of MDDs in ILD management [12, 13]. Compared to individual physician diagnoses, MDD enhances diagnostic confidence and inter-observer agreement between ILD specialists [14, 15].

Several prospective ILD registries have been initiated in North America and Europe [16–18]. Well-designed patient registries can provide real-world data on clinical practice, outcomes, safety, cost-effectiveness and guideline compliance. They elucidate the disease course and inform clinical decision-making [19–21]. However, few epidemiological studies on ILDs exist in Asia [22]. In addition, many registries focus solely on IPF, with limited data on other fibrotic ILDs like CTD-ILDs and non-IPF disorders [23]. The gender–age–physiology (GAP) score, modified Medical Research Council (mMRC) dyspnea scale, ventilatory efficiency slope, six-minute walk test (6MWT) distance, DLCO, and arterial CO2 pressure at maximal exercise may be predictors of mortality [24–26]. The SENSCIS [27] and INBUILD [28] trials showed the benefits of treatment with antifibrotics for systemic sclerosis-ILD, CTD-ILDs, and progressive non-IPF fibrotic ILDs. Thus, registries that include both IPF and non-IPF patients are warranted. Identification of prognostic factors across fibrotic lung diseases is critical for early introduction of antifibrotics and rehabilitation [29, 30].

The Registry of Interstitial Lung Diseases (REGILD) is a prospective, single-center registry study enrolling IPF and non-IPF populations in central Taiwan. Every patient enrolled in the REGILD registry is evaluated by MDD experts, including 3 pulmonologists, 3 rheumatologists, 2 radiologists, and 2 pathologists. CTD-ILD patients constitute a large cohort among non-IPF patients in the REGILD. Thus, our objective was to examine both common and unique long-term mortality predictors in IPF and CTD-ILD patients within a real-world prospective setting, emphasizing the significance of Multidisciplinary Discussion (MDD) in F-ILD diagnosis.

## Methods

### Study design, patient enrollment, and ethics

The REGILD is a prospective, single-center registry study that includes newly diagnosed patients with F-ILD, encompassing both IPF and non-IPF ILD patients. It is based at the Integrated Care Center for ILD at Taichung Veterans General Hospital, a tertiary referral center in Taiwan. Between December 2018 and December 2019, patients with F-ILDs confirmed with high-resolution computed tomography (HRCT) were enrolled after review by a multidisciplinary (MDD) team of expert pulmonologists, rheumatologists, radiologists, and pathologists. IPF patients were categorized as “definite,” “probable,” or “indeterminate” for IPF according to American Thoracic Society/European Respiratory Society/Japanese Respiratory Society/Latin American Thoracic Association guidelines [31]. F-ILD patients with diagnoses of established connective tissue disease were classified as CTD-ILD. Patients meeting criteria for interstitial pneumonia with autoimmune features (IPAF) were classified as IPAF.^13^ The exclusion criteria were age < 20 years and the presence of HIV infection. All enrolled patients provided written informed consent. This study complied with the Declaration of Helsinki and was approved by Taichung Veterans General Hospital Ethics Committee (approval number CE18325B, December 18, 2018) (Additional file 1: Fig. S1).

### Protocol for assessment of ILD in the REGILD cohort

The index day was defined as the day the patient signed the informed consent form. On the same day, participants completed the mMRC dyspnea scale questionnaire. Within 1 week of enrollment, participants underwent pulmonary function testing (PFT) and the 6MWT. Baseline demographic data, including age and gender, were recorded. The clinical data collected comprised presenting symptoms, physical examination findings, and antifibrotic medication use. The GAP score was calculated for each patient [3].

### PFT and 6MWT procedure

Forced vital capacity (FVC) and the DLCO were obtained from spirometry results according to the recommendations of the American Thoracic Society [32]. The 6MWT was performed according to the guidelines of the American Thoracic Society [33]. The patients were instructed to walk as far as possible within 6 min between two orange traffic cones placed 30 m apart in a corridor. Data on oxygen saturation (SpO2) and the distance walked in 6 min were obtained.

### Follow-up

The patients were followed up for mortality at yearly intervals. The cut-off date for death was February 1, 2023. The annual and overall incidences of mortality were recorded.

### Statistical analysis

Descriptive analysis was performed with absolute numbers and relative frequencies of categorical data. Continuous variables were presented as median (interquartile range [IQR]) for non-parametric data. The Mann–Whitney *U* test was used to compare continuous variables. The chi-square test and Fisher’s exact test were used to compare categorical variables. Univariate and multivariate Cox proportional hazards regression models were used to examine possible factors of mortality. Kaplan–Meier estimates and log-rank tests were used to calculate mortality rates. Statistical analyses were performed using SPSS Statistics (version 22; IBM Corporation, Armonk, NY, USA). A *p* value < 0.05 was considered statistically significant.

## Results

### Baseline characteristics and pulmonary physiology

Table 1 shows the characteristics of the patients in the registry. One hundred and four participants were enrolled: 33 (31.7%) were IPF and 71 (68.3%) were CTD-ILD. Their median age was 63 (IQR 58–69.8) years; most (61.5%) were females, and the majority (58.7%) had a definite usual interstitial pneumonia (UIP) pattern. The cohort had a median GAP score of 3 (IQR 1–3) and their median 6MWT was 446 (IQR 379.5–504.8) meters. Only 32 (30.8%) patients received antifibrotic medications. During a median follow-up duration of 4.1 (IQR 3.4–4.4) years, the annual mortality rate increased by about 6%, the 4-year mortality rate was 27.9%, and the overall mortality rate was 31.7%.

**Table 1:** Characteristics of the patient cohort

Charateristic^1^	Total (*n* = 104)	
Age	63	(58.0–69.8)
Male	40	(38.5%)
UIP pattern of HRCT	61	(58.7%)
Probable UIP	30	(39.3%)
Definite UIP	37	(60.7%)
Classification of ILD		
IPF	33	(31.7%)
CTD-ILD	71	(68.3%)
GAP score	3	(1–3)
mMRC dyspnea score	1	(0–2)
Anti-fibrotic agents	32	(30.8%)
Pulmonary function test		
FVC (L)	2.1	(1.7–2.7)
FVC (% predicted)	76.5	(61.0–88.8)
FEV1 (% predicted)	78.0	(61.0–88.0)
FEV1/FVC (%)	82.5	(78.0–86.0)
DL_CO_ (% predicted)	68.5	(50.8–81.3)
6MWT		
Distance (m)	446	(379.5–504.8)
Initial SpO2	96	(95.0–97.0)
SpO2 after 6MWT	89	(85.0–92.0)
Nadir	85.5	(79–89)
Nadir SpO2 < 90%	50	(53.2%)
Mortality		
Overall	33	(31.7%)
1-year	12	(11.5%)
2-year	16	(15.4%)
3-year	22	(21.2%)
4-year	29	(27.9%)
Follow-up time (year)	4.1	(3.4–4.4)

^1^Median (IQR); n (%)

^*^ IPF, idiopathic pulmonary fibrosis; UIP, usual interstitial pneumonia; HRCT, high-resolution computed tomography; GAP, gender–age–physiology; mMRC, modified Medical Research Council; FVC, forced vital capacity; FEV1, forced expiratory volume in one second; DLCO, diffusing capacity for carbon monoxide; 6MWT, six-minute walk test; SpO_2_, oxygen saturation

Table 2 compares demographic characteristics of IPF patients and CTD-ILD patients. Compared to patients with CTD-ILD, patients with IPF were older and more often male. In our registry, among patients without IPF, the most common CTD was idiopathic inflammatory myositis (35.5%), followed by primary systemic sclerosis (29.0%) and IPAF (12.7%). The proportion with UIP patterns on HRCT scans was higher in the IPF (81.8%) than in the CTD-ILD (47.9%) group (*p* = 0.002). This was refined with 14.1% (10 out of 71) and classified as a definite UIP pattern and 33.8% (24 out of 71) to a probable UIP pattern in accordance with the latest guidelines [3]. The median GAP score of the IPF group was 3 (IQR = 2–4.5), compared to 2 of the CTD-ILD group (IQR = 1–3, *p* < 0.001). The mMRC dyspnea score was higher in the IPF group than in the CTD-ILD group (1 [IQR = 1–3] vs. 1 [IQR = 0–1], *p* = 0.014). The proportion of patients receiving antifibrotic agents was higher in the IPF group than in the CTD-ILD group (60.6% vs. 16.9%,* p* < 0.001).

**Table 2:** Comparison of characteristics of patients with IPF and patients with CTD-ILD

Characteristic^1^	IPF (*n* = 33)		CTD-ILD (*n* = 71)		*p* value^2^
Age	69.0	(62–74)	61.0	(51–66)	< 0.001
Male	24	(72.7%)	16	(22.5%)	< 0.001
Definite UIP pattern	27	(81.8%)	34	(47.9%)	0.002
Pattern in HRCT					< 0.001
NSIP pattern	0	(0%)	37	(52.1%)	
UIP pattern (Probable)	6	(18.2%)	24	(33.8%)	
UIP pattern (Definite)	27	(81.8%)	10	(14.1%)	
GAP score	3.0	(2–4.5)	2.0	(1–3)	< 0.001
mMRC dyspnea score	1.0	(1–3)	1.0	(0–1)	0.014
Anti-fibrotic agents	20	(60.6%)	12	(16.9%)	< 0.001
Pulmonary function test					
FVC (L)	2.2	(1.8–2.8)	2.0	(1.7–2.6)	0.451
FVC (% predicted)	80.0	(61–96)	74.0	(60–88)	0.550
FEV1 (% predicted)	81.0	(64–91.5)	77.0	(59–87)	0.287
FEV1/FVC (%)	84.0	(78.5–89.5)	82.0	(78–85)	0.033
DL_CO_ (% predicted)	64.0	(39–79)	71.0	(53.5–82.5)	0.112
6MWT					
Distance (m)	403.5	(345–462)	461.0	(399–516.8)	0.007
Initial SpO_2_	96.0	(93.8–97)	96.0	(95–97)	0.168
SpO_2_ after 6MWT	88.0	(83.3–92)	89.5	(85–92)	0.365
Nadir	84.0	(79–89)	86.0	(79–89)	0.711
Nadir SpO_2_ < 90%	18	(60.0%)	32	(50.0%)	0.494
Mortality					
1-year	5	(15.2%)	7	(9.9%)	0.513
2-year	9	(27.3%)	7	(9.9%)	0.022
3-year	14	(42.4%)	8	(11.3%)	< 0.001
4-year	17	(51.5%)	12	(16.9%)	< 0.001
Overall	20	(60.6%)	13	(18.3%)	< 0.001
Follow-up time (year)	3.4	(1.9–4.3)	4.2	(3.7–4.4)	0.002

^1^Median (IQR); n (%)

^2^Mann–Whitney U test, chi-square test, Fisher’s exact test

^*^ IPF, idiopathic pulmonary fibrosis; UIP, usual interstitial pneumonia; HRCT, high-resolution computed tomography; GAP, gender–age–physiology; mMRC, modified Medical Research Council; FVC, forced vital capacity; FEV1, forced expiratory volume in one second; DLCO, diffusing capacity for carbon monoxide; 6MWT, six-minute walk test; SpO_2_, oxygen saturation

In this cohort, baseline FVC, forced expiratory volume in one second (FEV1), and DLCO (% predicted) data did not differ significantly between groups. The CTD-ILD group had greater 6MWT distances than the IPF group. SpO_2_ before and after the 6MWT did not differ significantly between the IPF and CTD-ILD groups. More than half of the participants in both groups had a nadir SpO_2_ of < 90%. At the end of follow-up, 60.6% of IPF patients were deceased compared to only 18.3% of CTD-ILDs patients (*p* < 0.001).

### Primary outcome of overall mortality

Of the 104 patients, 33 died by the cutoff date of February 1, 2023; 20 had IPF and 13 had CTD-ILD (Table 3). Older age, male sex, IPF, UIP pattern, high GAP score, high mMRC score, and use of pulmonary fibrosis drugs were associated with higher mortality (*p* < 0.001). The long-term mortality of patients with lower FEV1/FVC, shorter 6-min walking distance, and lower initial and final SpO_2_ was higher (*p* < 0.05; Table 3).

**Table 3:** Comparison of characteristics of surviving patients and deceased patients

Characteristic^1^	Surviving (*n* = 71)		Deceased (*n* = 33)		*p* value^2^
Age	60.0	(52–67)	69.0	(63–72.5)	< 0.001
Male	17	(23.9%)	23	(69.7%)	< 0.001
Classification of ILD					< 0.001
IPF	13	(18.3%)	20	(60.6%)	
CTD-ILD	58	(81.7%)	13	(39.4%)	
UIP pattern	33	(46.5%)	28	(84.8%)	< 0.001
GAP score	2.0	(1–3)	4.0	(3–5)	< 0.001
mMRC dyspnea score	1.0	(0–1)	2.0	(1–3)	< 0.001
Anti-fibrotic agents	14	(19.7%)	18	(54.5%)	< 0.001
Pulmonary function test					
FVC (L)	77.0	(66–92)	67.0	(55.5–87.5)	0.194
FVC (% predicted)	79.0	(65–88)	71.0	(54.5–88)	0.353
FEV1 (% predicted)	82.0	(78–85)	85.0	(76.5–89.5)	0.071
FEV1/FVC (%)	73.0	(60–85)	54.0	(37–64)	< 0.001
DL_CO_ (% predicted)	2.2	(1.9–2.7)	1.9	(1.5–2.7)	0.211
6MWT					
Distance (m)	462.0	(399.8–520.8)	391.0	(345–447.8)	0.001
Initial SpO_2_	97.0	(95–97)	96.0	(92–96)	0.010
SpO_2_ after 6MWT	90.0	(86.3–92)	87.0	(78.8–92)	0.030
Nadir SpO_2_ < 90%	33	(48.5%)	17	(65.4%)	0.143

IPF, idiopathic pulmonary fibrosis; UIP, usual interstitial pneumonia; HRCT, high-resolution computed tomography; GAP, gender–age–physiology; mMRC, modified Medical Research Council; FVC, forced vital capacity; FEV1, forced expiratory volume in one second; DLCO, diffusing capacity for carbon monoxide; 6MWT, six-minute walk test; SpO_2_, oxygen saturation

^1^Median (IQR); n (%)

^2^Mann–Whitney U test, chi-square test, Fisher’s exact test

**Fig. 1:**
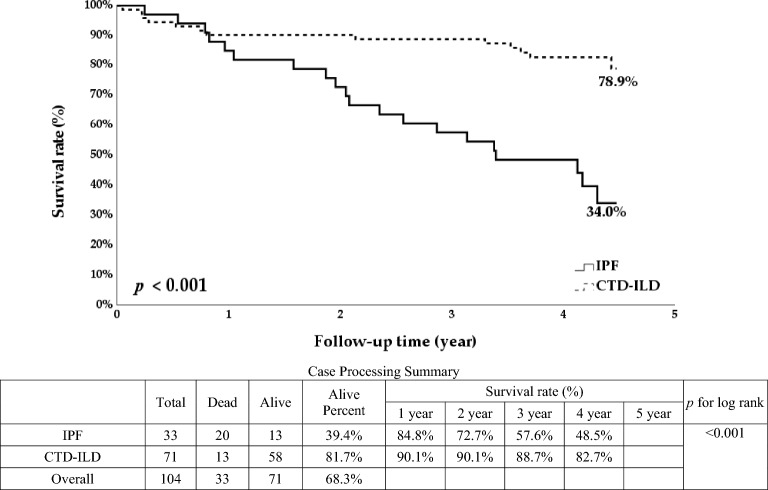
Kaplan–Meier survival curve according to the interstitial lung disease condition

**Fig. 2:**
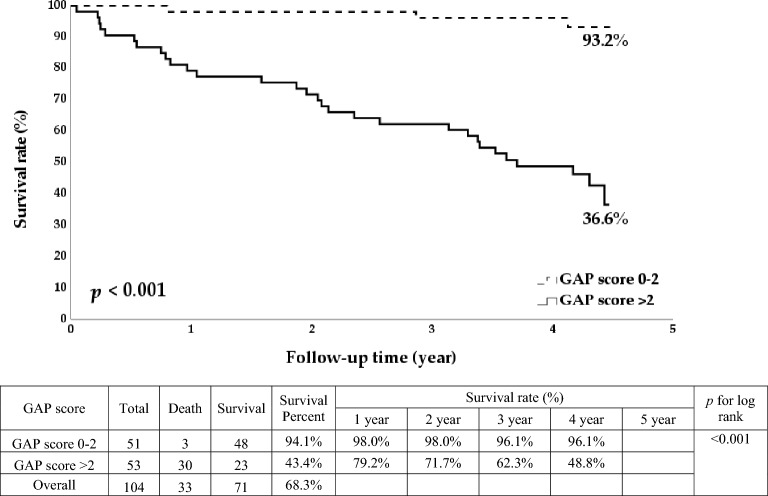
Kaplan–Meier survival curve according to the gender–age–physiology (GAP) score

**Fig. 3:**
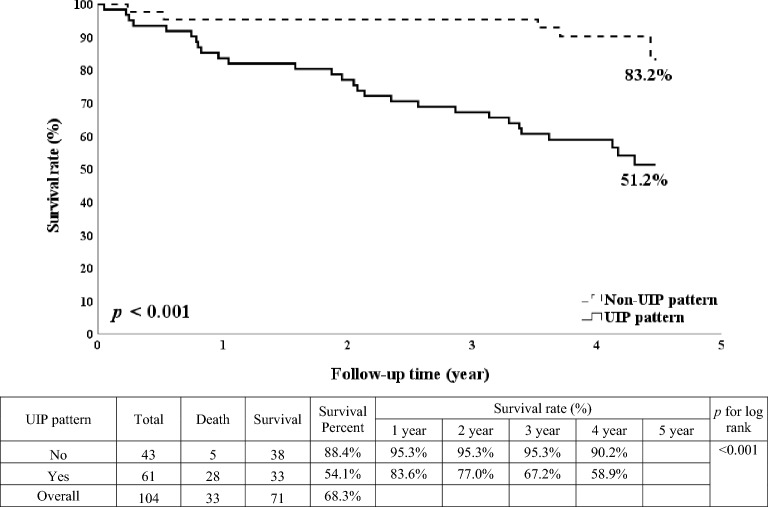
Kaplan–Meier survival curve according to the usual interstitial pneumonia (UIP) pattern

The overall survival rate of CTD-ILD patients was more than twice that of IPF patients (78.9% vs. 34%, *p* < 0.001; Fig. 1). The overall survival rate of patients with a GAP score of 0–2 was also significantly higher than that of patients who scored > 2 (93.2% vs. 36.6%, *p* < 0.001; Fig. 2). Likewise, the overall survival rate of patients with a non-definite UIP pattern was significantly higher than that of patients with a definite UIP pattern (83.2% vs, 51.2%, *p* < 0.001; Fig. 3).

### Univariate and multivariate analyses of factors associated with overall mortality

Cox proportional hazards regression analysis of mortality is shown in Table 4. In univariate analyses, higher age, male gender, IPF, definite UIP pattern, higher GAP score, higher mMRC dyspnea score, lower DLCO (% predicted), lower 6MWT distance, lower initial SpO_2_, lower SpO_2_ after 6MWT, and lower nadir SpO_2_ were significantly associated with mortality. In the multivariate analysis, only a GAP score > 2 (hazard ratio [HR] 16.7, 95% CI 3.28–85.14, *p* = 0.001) and definite UIP pattern (HR 4.08, 95% CI 1.07–15.5, *p* = 0.039) were significantly associated with overall mortality.

**Table 4:** Univariate and multivariate analyses of factors associated with overall mortality

Characteristic^1^	Simple model			Multiple model			Multiple model		
	HR	(95% CI)	*p* value	HR	(95% CI)	*p* value	HR	(95% CI)	*p* value^2^
Age	1.05	(1.02–1.08)	0.003						
Male	4.70	(2.23–9.91)	< 0.001						
IPF vs CTD-ILD	4.33	(2.14–8.77)	< 0.001	0.50	(0.20–1.26)	0.140	0.59	(0.22–1.59)	0.298
UIP pattern	4.95	(1.91–12.84)	0.001	3.73	(0.94–14.72)	0.061	4.08	(1.07–15.50)	0.039
GAP score	1.75	(1.47–2.10)	< 0.001	2.49	(1.61–3.86)	< 0.001			
GAP score > 2	13.61	(4.14–44.71)	< 0.001				16.70	(3.28–85.14)	0.001
mMRC dyspnea score	1.81	(1.38–2.38)	< 0.001	1.10	(0.62–1.95)	0.746	1.33	(0.76–2.31)	0.320
Pulmonary function									
FVC (% predicted)	0.99	(0.98–1.01)	0.401						
FEV1 (%)	1.00	(0.98–1.02)	0.956						
FEV1/FVC (%)	1.06	(1.00–1.12)	0.049						
DL_CO_ (% predicted)	0.96	(0.94–0.98)	< 0.001	1.02	(1.00–1.05)	0.104	1.01	(0.99–1.04)	0.335
6MWT									
Distance (m)	0.99	(0.99–0.997)	< 0.001	1.00	(0.99–1.01)	0.887	1.00	(1.00–1.01)	0.672
Initial SpO_2_	0.74	(0.62–0.87)	< 0.001	0.80	(0.63–1.03)	0.090	0.88	(0.67–1.16)	0.364
SpO_2_ after 6MWT	0.94	(0.89–0.98)	0.008	0.95	(0.87–1.03)	0.231	0.91	(0.81–1.03)	0.152
Nadir SpO_2_	0.89	(0.81–0.97)	0.007				1.01	(0.90–1.14)	0.831
Nadir SpO_2_ < 90%	1.83	(0.81–4.11)	0.144						

IPF, idiopathic pulmonary fibrosis; UIP, usual interstitial pneumonia; HRCT, high-resolution computed tomography; GAP, gender–age–physiology; mMRC, modified Medical Research Council; FVC, forced vital capacity; FEV1, forced expiratory volume in one second; DLCO, diffusing capacity for carbon monoxide; 6MWT, 6-min walk test; SpO_2_, oxygen saturation

^1^Hazard ratio (HR) and 95% confidence interval (95% CI)

^2^Cox proportional hazards regression

## Discussion

In this prospective cohort study, we recorded baseline clinical, physiological, and radiological characteristics of patients with IPF and CTD-ILDs and followed them up for more than four years. The long-term mortality rates of patients with IPF, definite UIP pattern, and a GAP score of > 2 were significantly higher than those of patients with CTD-ILDs, non-definite UIP pattern, and a GAP score of 0–2. Nevertheless, in the multivariate analysis of factors potentially associated with mortality, the GAP score and definite UIP pattern were the only significant predictors of overall mortality.

Although the study cohort had relatively advanced disease, as indicated by the GAP score, PFT, and 6MWT parameters, only one third of the cohort, most of them belonging to the IPF group, received antifibrotic medications. This may explain the paradoxically higher proportion of antifibrotic receivers (54.5%) in the mortality group. The early start of antifibrotic medication for ILD patients is of fundamental importance. The rate of decline in lung function was similar in both IPF groups with preserved or reduced lung function; antifibrotic treatment was effective in both groups. Therefore, antifibrotic medications should be started for IPF, irrespective of symptoms or lung function [34]. The use of antifibrotic medications to treat many non IPF-ILDs was recently recommended [34]. In Taiwan, two antifibrotic medications—nintedanib and pirfenidone—are currently reimbursed by National Health Insurance for patients with IPF and interstitial lung disease associated with systemic sclerosis [35].

In our study, the long-term mortality of patients with IPF was higher than that of patients with CTD-ILD, confirming previous research showing worse prognosis of IPF [36]. The median survival of patients with IPF is 2–5 years after diagnosis [3], while CTD-ILD patients tend to have more favorable outcomes [36]. The mechanisms underlying this difference in mortality remain unclear but are likely to be related to differences in pathogenesis between IPF and CTD-ILD [36].

Importantly, our study demonstrated that the GAP score and UIP pattern on imaging are significant predictors of long-term mortality in both IPF and CTD-ILD. These common prognostic factors could be applied to ILD patients regardless of whether they have IPF or CTD-ILD. The GAP model predicting mortality risk based on gender, age, and physiology was originally developed for IPF [3]. However, previous studies and our findings show that the GAP score is also a predictor of mortality across other ILD subtypes including CTD-ILD [37]. On the other hand, the UIP pattern predicts worse prognosis in both IPF and CTD-ILD [38, 39]. That the GAP score and UIP pattern can be used in prognostication for all ILD patients underscores key commonalities in disease behavior between IPF and CTD-ILD.

Identification of these shared mortality predictors is crucial because it could prompt earlier interventions like treatment with antifibrotics or enrollment in pulmonary rehabilitation across ILD subtypes. Such measures could potentially prolong patients’ lives. Specifically, physicians should closely monitor the results of function tests like DLCO for decreases signifying progression, even in patients with CTD-ILD. Declines in physiology scores factor into worsening GAP scores; therefore, maintaining function is imperative. With further validation, these predictors could be incorporated into clinical decision tools guiding screening, follow-up, and treatment protocols to improve patient outcomes.

Our study has limitations that warrant further consideration. As a single-center study with only 104 patients, its sample size and generalizability are limited. Inclusion of longitudinal PFT and radiological data could have provided deeper insights into mortality predictors. Nonetheless, our findings offer a useful perspective on long-term prognostication in fibrotic ILDs. Moving forward, larger multicenter collaborations, such as the PROgressive Fibrosing lung diseasES.network, may enable more robust prognostic modeling.

## Conclusion

The long-term mortality rate of IPF patients was higher than that of CTD-ILD patients. The GAP score and UIP patterns were significant mortality predictors for both IPF and CTD-ILD patients. While validation of these findings with larger cohorts is warranted, they could help physicians and decision-makers to take more effective steps, such as earlier start of antifibrotic medications, for managing ILD patients.

### Supplementary Information

**Additional file 1**. Study flowchart outlining the total number of cases during the study period.

## Data Availability

The datasets used and/or analyzed during the current study are available from the corresponding author on reasonable request.

## Abbreviations

6MWT: 6-Minute walking test
CTD-ILD: Connective tissue disease-associated interstitial lung disease
DLCO: Diffusing capacity for carbon monoxide
F-ILD: Fibrotic interstitial lung disease
GAP: Gender–age–physiology
HR: Hazard ratio
HRCT: High-resolution computed tomography
IPF: Idiopathic pulmonary fibrosis
IQR: Interquartile range
IPAF: Interstitial pneumonia with autoimmune features
FEV1/FVC: Lower forced expiratory volume in one second/forced vital capacity
mMRC: Modified Medical Research Council
MDD: Multidisciplinary discussion
REGILD: Registry of Interstitial Lung Diseases
UIP: Usual interstitial pneumonia
