# Supplementary material for: Factors influencing long-term outcomes in fibrotic interstitial lung disease (F-ILD) diagnosed through multidisciplinary discussion (MDD): a prospective cohort study
Source: Eur J Med Res. 2024 Jan 30;29:91. doi: 10.1186/s40001-024-01673-2 (PMC10826228; doi:10.1186/s40001-024-01673-2)
Supplement: Supplementary file 1 — Additional file 1. Study flowchart outlining the total number of cases during the study period. [file 40001_2024_1673_MOESM1_ESM.docx]

**Table 1.** Characteristics of the patients in the registry. One hundred and four participants were enrolled: 33 (31.7%) were IPF and 71 (68.3%) were CTD-ILD. Their median age was 63 (IQR = 58-69.8) years; most (61.5%) were females, and the majority (58.7%) had a definite usual interstitial pneumonia (UIP) pattern. The cohort had a median GAP score of 3 (IQR = 1-3) and their median 6MWT was 446 (IQR = 379.5-504.8) meters. Only 32 (30.8%) patients received antifibrotic medications. During a median follow-up duration of 4.1 (IQR = 3.4-4.4) years, the annual mortality rate increased by about 6%, the four-year mortality rate was 27.9%, and the overall mortality rate was 31.7%. **Table 2.** Comparison of the demographic characteristics of IPF patients and CTD-ILD patients. Compared to patients with CTD-ILD, patients with IPF were older and more often male. In our registry, among patients without IPF, the most common CTD was idiopathic inflammatory myositis (35.5%), followed by primary systemic sclerosis (29.0%) and IPAF (12.7%). The proportion with UIP patterns on HRCT scans was higher in the IPF (81.8%) than in the CTD-ILD (47.9%) group (*p* = 0.002). This was refined with 14.1% (10 out of 71) and classified as a definite UIP pattern and 33.8% (24 out of 71) to a probable UIP pattern in accordance with the latest guidelines. The median GAP score of the IPF group was 3 (IQR = 2-4.5), compared to 2 of the CTD-ILD group (IQR = 1-3, *p* < 0.001). The mMRC dyspnea score was higher in the IPF group than in the CTD-ILD group (1 [IQR = 1-3] vs. 1 [IQR = 0-1], *p* = 0.014). The proportion of patients receiving antifibrotic agents was higher in the IPF group than in the CTD-ILD group (60.6% vs. 16.9%, *p* < 0.001). **Table 3.** Of the 104 patients, 33 died by the cutoff date of February 1, 2023; 20 had IPF and 13 had CTD-ILD. Older age, male sex, IPF, UIP pattern, high GAP score, high mMRC score, and use of pulmonary fibrosis drugs were associated with higher mortality (*p* < 0.001). The long-term mortality of patients with lower FEV1/FVC, shorter six-minute walking distance, and lower initial and final SpO_2_ was higher (*p* < 0.05). **Table 4.** Cox proportional hazards regression analysis of mortality. In univariate analyses, higher age, male gender, IPF, definite UIP pattern, higher GAP score, higher mMRC dyspnea score, lower DLCO (% predicted), lower 6MWT distance, lower initial SpO_2_, lower SpO_2_ after 6MWT, and lower nadir SpO_2_ were significantly associated with mortality. In the multivariate analysis, only a GAP score >2 (hazard ratio [HR] = 16.7, 95% CI = 3.28-85.14, *p* = 0.001) and definite UIP pattern (HR = 4.08, 95% CI = 1.07-15.5, *p* = 0.039) were significantly associated with overall mortality.
